# Sea Cucumber-Derived Peptides Alleviate Oxidative Stress in Neuroblastoma Cells and Improve Survival in *C. elegans* Exposed to Neurotoxic Paraquat

**DOI:** 10.1155/2021/8842926

**Published:** 2021-04-19

**Authors:** Meng Lu, Ajay Mishra, Chiara Boschetti, Jing Lin, Yushuang Liu, Hongliang Huang, Clemens F. Kaminski, Zebo Huang, Alan Tunnacliffe, Gabriele S. Kaminski Schierle

**Affiliations:** ^1^Cambridge Infinitus Research Centre, Department of Chemical Engineering and Biotechnology, University of Cambridge, West Cambridge Site, Philippa Fawcett Drive, Cambridge CB3 0AS, UK; ^2^Research Institute for Food Nutrition and Human Health, School of Food Science and Engineering, South China University of Technology, Guangzhou 510640, China; ^3^School of Biosciences and Biopharmaceutics, Guangdong Pharmaceutical University, Guangzhou 510006, China

## Abstract

Oxidative stress results when the production of oxidants outweighs the capacity of the antioxidant defence mechanisms. This can lead to pathological conditions including cancer and neurodegeneration. Consequently, there is considerable interest in compounds with antioxidant activity, including those from natural sources. Here, we characterise the antioxidant activity of three novel peptides identified in protein hydrolysates from the sea cucumber *Apostichopus japonicus*. Under oxidative stress conditions, synthetic versions of the sea cucumber peptides significantly compensate for glutathione depletion, decrease mitochondrial superoxide levels, and alleviate mitophagy in human neuroblastoma cells. Moreover, orally supplied peptides improve survival of the *Caenorhabditis elegans* after treatment with paraquat, the latter of which leads to the production of excessive oxidative stress. Thus, the sea cucumber peptides exhibit antioxidant activity at both the cellular and organism levels and might prove attractive as nutritional supplements for healthy ageing.

## 1. Introduction

Loss of redox homeostasis, due to an imbalance between the production and neutralisation of reactive oxygen species (ROS), leads to oxidative stress in cells [1, 2]. Cellular ROS are produced as a result of normal intracellular metabolic reactions in organelles such as mitochondria and peroxisomes, as well as by the activity of cytosolic enzymes such as lipoxygenase, NADPH oxidase, and cytochrome P450 [1, 2]. ROS can also be produced in cells by external factors, including exposure to irradiation, chemicals, or environmental toxins. Excess levels of ROS can damage DNA, proteins, and lipids, leading to cellular dysfunction and finally leading to disease [3]. To combat and regulate elevated levels of ROS, animals and plants have evolved sophisticated enzymatic and nonenzymatic antioxidant defence systems, like catalase (CAT), superoxide dismutase (SOD), and glutathione (GSH) [1]. However, ageing can lead to a decline of endogenous antioxidants, and the concomitant increase in ROS or free radical levels can lead to age-related pathologies, such as cardiovascular disease and neurodegeneration [4–8]. Therefore, the identification, development, and use of exogenous antioxidants represent a possible strategy to combat the above-listed diseases [9]. Indeed, due to their physiological and pharmacological activities, natural bioactive substances, such as peptides [10–12], polysaccharides [13], and polyphenols [14], have gained increased attention in health care and longevity research and, more specifically, have been shown to alleviate oxidative stress and the progression of age-related diseases.

Peptides are an attractive class of compounds to screen for novel antioxidant compounds because of their stability, safety, bioavailability, and tolerability. A variety of biologically active peptides have been discovered from diverse natural resources. Holothurians are echinoderms from the class Holothuroidea. In China, Holothurians, known as sea cucumbers, have been used as a food supplement for more than 2000 years [15]. Previous research has also demonstrated a variety of bioactive compounds in different sea cucumbers [16], containing antiangiogenic [17] and antioxidant [18] properties. In particular, scientists have successfully isolated peptides from Apostichopus japonicus (A. japonicus) and demonstrated their antioxidant activities in *in vitro* scavenging assays against hydroxyl radicals and superoxide anions [18]. Peptides extracted from the *A. japonicus* gonad have been purified and found to inhibit the angiotensin-converting enzyme, and thus, the extracted sea cucumber extract may facilitate the treatment of cardiovascular diseases [17]. However, whether the peptides derived from *A. japonicus* maintain their bioactivity in cells and if so how they help the cells to cope with oxidative stress are largely unknown.

In a recent study, we hydrolysed proteins from *A. japonicus* by enzymatic hydrolysis and tested the antioxidant and antiageing activities using nematode *Caenorhabditis elegans*, a commonly used model in the study of longevity [19]. The hydrolysates were then purified, and the constituent peptides comprising antioxidant activity were identified and sequenced [19]. To specifically analyse the antioxidant effects of individual peptides with known sequences rather than a mixture of different peptides, we generated synthetic versions of individual sea cucumber peptides and tested them in both human cells and nematodes using various experimental approaches. First, our results demonstrated strong antioxidant activity of the peptides in human neuroblastoma cells (SH-SY5H) after exposure to H_2_O_2_. Further investigations revealed that the peptides can largely reduce superoxide radicals in mitochondria and protect mitochondria from superoxide-induced mitophagy. Finally, we provide evidence that sea cucumber peptides fed orally to *C. elegans* can also improve survival of worms after exposure to paraquat.

## 2. Materials and Methods

### 2.1. Cell Culture

Human neuroblastoma cells (SH-SY5Y) were purchased from the European Collection of Cell Cultures (ECACC, Sigma-Aldrich) and were grown in a 1 : 1 minimal essential medium (MEM) (Sigma-Aldrich) and nutrient mixture F-12 Ham (Sigma-Aldrich) supplemented with 15% FBS (Gibco), 1% nonessential amino acids, 2°mM GlutaMAX, and 1% antibiotic-antimycotic (all Thermo Fisher Scientific). Cells were grown in T75 or T25 flasks and incubated at 37°C 5% CO_2_. Cells were kept in a logarithmic growth phase and passaged when reaching 80–90% confluency (approximately every 3–4 days). The medium was changed every 3 days. Cell counting and viability assays were routinely carried out using a haemocytometer and trypan blue. Cells were negative for mycoplasma infection. For experiments, cells were plated in various treated cell culture dishes at an appropriate cell density according to requirements (see Results).

### 2.2. Peptide Synthesis and Analytical Mass Spectrometry

Identification and synthesis of peptides were performed as described previously [20]. The peptides were synthesised by Shanghai Top-peptide Biotechnology Co., Ltd. (Shanghai, China) using solid-phase synthesis methods with a purity of >95%.

### 2.3. Cellular Toxicity Assays

The cellular toxicity of compounds was measured by performing colorimetric cell metabolic activity assays using either MTT or MTS assay kits (Promega) according to the manufacturer's protocol. Essentially, cells were seeded in 96-well plates at a density of 10^4^ cells in 100 *μ*l medium per well. After 24 h incubation at 37°C, cells were treated with peptides or a vehicle control at the indicated concentrations (see Results) and were further incubated for 72 h before the addition of MTS or MTT reagent. For the MTS assay, a 25 *μ*l MTS reagent was directly added to the culture medium in each well containing cells. The dish was incubated at 37°C for 2 h before the absorbance of the formazan dye at 506 nm was recorded using the Envision Multilabel plate reader. For the MTT assay, after 72 h cell treatment, culture medium was replaced by a mixture of 10 *μ*l MTT solution in PBS (5 mg/ml) and 90 *μ*l fresh medium. The plates were incubated for 4 h at 37°C, and then, the medium was replaced with 100 *μ*l DMSO to dissolve the MTT formazan. Finally, the absorbance of the formazan dye at 570 nm was measured by a plate reader.

### 2.4. Oxidative Stress Assay Using CM-H2DCFDA

SH-SY5Y cells were grown in 96-well cell culture dishes until they reached 60-70% confluency. Cells were treated with 50 *μ*M peptides and incubated for 24 h at 37°C. As a positive control, cells were also treated with 50 *μ*M glutathione (GSH). After incubation, cellular oxidative stress was induced by treating cells with 2 mM H_2_O_2_ for 3 h at 37°C. Cells were then washed three times with PBS before labelling them with 1 *μ*M CM-H2DCFDA (Invitrogen, C62827) for 30 min at 37°C. Fluorescence intensity in SH-SY5Y cells was measured using the Envision Multilabel plate reader at Ex/Em 492/527 nm wavelength. Statistical significance was determined by Tukey one-way ANOVA. ^∗∗^*P* < 0.01. Error bars show SD. Experiments were repeated three times.

### 2.5. Apoptotic and Necrotic Cell Death Assays

SH-SY5Y cells were grown in Lab-Tek dishes until they reached 60-70% confluency. Cells were treated with 50 *μ*M peptides and incubated for 24 h at 37°C. As a positive control, cells were also treated with 50 *μ*M glutathione (GSH). After incubation, cellular oxidative stress was induced by treating cells with 2 mM H_2_O_2_ for 3 h at 37°C. Cells were then washed three times with PBS before labelling them with 1 *μ*l Apopxin Green Indicator for apoptosis or 1 *μ*l 7-AAD for necrosis for 30 min at 37°C before imaging according to the manufacturer's protocol (Abcam, ab176749). 200 cells were counted for each condition in each experiment for three repeats. Statistical significance was determined by Tukey one-way ANOVA. ^∗∗∗∗^*P* < 0.0001, ^∗∗^*P* < 0.001, and ^∗∗^*P* < 0.01. Error bars show SD. Experiments were repeated three times.

### 2.6. Measurement of Intracellular GSH

To measure cellular GSH, the fluorogenic dye monochlorobimane (Sigma) was used as described [21]. Following treatment of cells for 24 h (see Results), SH-SY5Y cells were washed with PBS three times and incubated with 40 *μ*M monochlorobimane, dissolved in medium, for 1 h at 37°C. Cells were then washed twice with PBS and treated with 600 *μ*M ABAP to induce ROS production. One hour after the addition of ABAP, monochlorobimane fluorescence was recorded at Ex^405^/Em^486^ using the Envision Multilabel plate reader. Data were plotted using GraphPad Prism software. Two-way ANOVA and Dunnett's multiple comparison test were applied to measure the statistical significance of each treatment relative to the no-peptide control as well as to compare differences between pairs of peptide concentrations. ^∗∗∗∗^*P* < 0.0001, ^∗∗∗^*P* < 0.001, and ^∗∗^*P* < 0.01.

### 2.7. Measurement of Mitochondrial Superoxide Using MitoSOX Red

To measure the superoxide levels in mitochondria, MitoSOX Red (Thermo Fisher Scientific) fluorogenic dye was used. MitoSOX Red intensity was measured by both confocal microscopy and flow cytometry. For the imaging assay, SH-SY5Y cells were grown in glass-bottomed MatTek dishes (MatTek Corporation) until 50-70% confluency was reached. After peptide treatment and incubation (see Results), cells were washed three times with PBS before treatment with 2 mM H_2_O_2_ (Sigma) for 3 h. The H_2_O_2_ reaction was stopped by washing the cells three times with PBS. Subsequently, cells were labelled with 2 *μ*M MitoSOX Red dye for 30 min at 37°C before imaging the cells by confocal microscopy (Ex^510^/Em^580^). For flow cytometry experiments, cells were grown and treated as described above, except the cells were grown in 24-well cell culture dishes. After labelling the cells with MitoSOX Red, they were washed with PBS, harvested by trypsinisation, and resuspended in PBS at no more than 1000 cells/*μ*l. MitoSOX Red fluorescence intensity was quantified by analytical flow cytometry (Guava, Merck). Statistical significance was determined by Tukey one-way ANOVA. ^∗∗∗^*P* < 0.001, ^∗∗^*P* < 0.01, and ^∗^*P* < 0.05; ns: not significant. Bar charts were produced using GraphPad Prism 6 software.

To visualise colocalisation of peptides and lysosomes, we used our custom-built structured illumination microscope (SIM) providing a spatial resolution approaching 90 nm at frame rates reaching 22 Hz [22]. The cells were grown in glass-bottomed Petri dishes at 37°C in a 5% CO_2_ atmosphere. On the day of imaging, Petri dishes were first stabilised in the incubation chamber of the SIM system with continuous air supply (37°C and 5% CO_2_). Hardware control and image reconstruction were performed with software written in LabView and Matlab [23]. For motion analysis and visualisation, ImageJ was used.

### 2.8. Manders' Coefficient to Quantify the Colocalisation of Mitochondria and Lysosomes

Live samples were imaged on a confocal microscope (Leica TCS SP5, using the Leica Application Suite (LAS AF)) with a HCX PL APO 40x/1.25-0.75 oil objective lens (Leica) or HCX PL APO 60x/1.40-0.60 oil objective lens (Leica) at room temperature. Fluorochromes used for individual experiments are stated in the figure legends. To quantify the degree of mitophagy induced by the accumulation of superoxide in mitochondria, we stained the SH-SY5Y cells with 1 *μ*M SiR-Lysosome and 1 *μ*M verapamil (Spirochrome, CY-SC012), which stayed in the medium during the imaging experiment. Cells were incubated with 1 *μ*M rhodamine B for 30 min at 37°C and 5% CO_2_ and washed three times with PBS before imaging. Images collected by confocal microscopy were analysed using the Fiji plugin Coloc2 (https://imagej.net/Coloc_2), and Manders' coefficient was used to quantify the degree of colocalisation between mitochondria and lysosomes. Statistical significance was determined by Tukey one-way ANOVA. ^∗∗∗^*P* < 0.001, ^∗∗^*P* < 0.01, and ^∗^*P* < 0.05; ns: not significant. Bar charts were produced using GraphPad Prism 6 software.

### 2.9. Paraquat Survival Assay in *C. elegans*

The oxidative survival assay using paraquat was performed in wild-type *C. elegans* as described previously [24]. Briefly, synchronized L1 larvae were placed in 96-well plates at a density of 25-30 worms per well in S medium containing *E. coli* NA22 as food and incubated at 20°C with shaking for 42-45 h until they reached the L4 stage. The L4 worms were then washed with S medium, transferred to 96-well plates at a density of 15-20 worms per well with bacterial food and 75 *μ*g/ml 5-fluoro-2′-deoxyuridine, and treated with peptides at final concentrations of 1.0, 2.0, and 4.0 mM at 20°C for 24 h with shaking. Next, 100 mM paraquat was applied to each well,, and the culture plates were incubated for another 24 h at 20°C in a shaking incubator. After paraquat exposure, the numbers of live and dead worms were scored every 12 h until all the worms were dead.

## 3. Results

### 3.1. Sea Cucumber-Derived Peptides Are Nontoxic and Have Antioxidant Activity as They Protect Cells Against Hydrogen Peroxide

**Figure 1:**
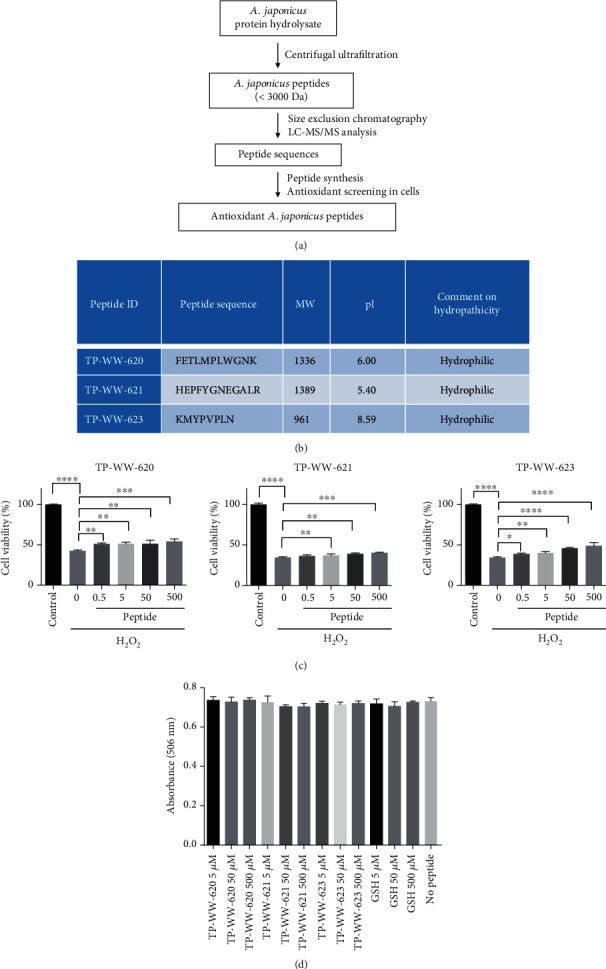
Sea cucumber-derived peptides are nontoxic and protect cells against hydrogen peroxide. (a) A protein hydrolysate from *A. japonicus* was subjected to centrifugal ultrafiltration to separate peptides less than 3000 Da. The purified pool was further subjected to size exclusion chromatography and LC-MS/MS analysis to identify peptide sequences. The identified peptide sequences were chemically synthesised and subjected to functional screening to identify antioxidant peptides. (b) Sequences and biophysical properties of peptides. (c) SH-SY5Y cells were plated at a density of 1 × 10^4^ cells per well in 96-well plates and grown to approximately 70%-80% confluency. Cells were then treated with different concentrations of peptides for 24 h at 37°C before being washed once with PBS. Next, the cells were incubated with 600 *μ*M H_2_O_2_ for 4 h without peptides at 37°C. The metabolic activity of cells was measured by the MTT assay using a plate reader to record absorbance at 570 nm. Metabolic activity is plotted as percentages relative to the untreated control group, which is denoted as 100%. Statistical significance was determined by Tukey one-way ANOVA. Error bars show SD (*n* = 3). Experiments were repeated three times. (d) SH-SY5Y cells were grown in 96-well cell culture dishes until they reached 60-70% confluency. Cells were then treated with different concentrations of peptides as shown in the figure. After treatment, cells were incubated for 72 h at 37°C. Using a colorimetric MTS assay, absorbance at 506 nm was recorded using a plate reader to quantify cell proliferation.

We have previously identified novel peptide sequences from protein hydrolysates of sea cucumber (*A. japonicus*) using mass spectrometry [19]. To ensure that subsequent experiments were performed with pure material, we performed chemical synthesis of a subset of the identified peptides and tested these for antioxidant activity (Figure 1(a)). *In silico* analysis suggested that all three peptide sequences are hydrophilic in nature with GRAVY scores of -0.200, -1.125, and -0.250 for TP-WW620, TP-WW-621, and TP-WW-623, respectively (Figure 1(b)). Initially, we assessed the cell toxicity of the synthesised peptides using the neuroblastoma cell line, SH-SY5Y. Cells were treated with peptides for 72 h at 37°C, but we observed no toxicity within the concentration range used (Figure 1(d)). To test for antioxidant activity, we first investigated whether the peptides could protect SH-SY5Y cells exposed to H_2_O_2_. H_2_O_2_ is a well-known oxidant and is produced within cells during normal cell metabolism [25]. However, excessive H_2_O_2_ causes significant cell damage [26]. In this experiment, we plated cells in 96-well cell culture dishes for 24 h or more. The cells were then treated independently with the peptides TP-WW-620, TP-WW-621, and TP-WW-623 (Figure 1(b)) dissolved in culture medium, at concentrations of 0.5, 5.0, 50, and 500 *μ*M. After 24 h, peptides were removed by washing cells with PBS, after which 600 *μ*M H_2_O_2_ was added to the growth medium for 4 h at 37°C. Subsequently, cells were washed, and their metabolic rate was measured using a MTT assay. We found that, although cell metabolic activity was reduced by more than 50% in cells exposed to H_2_O_2_, this effect was significantly reduced in cells pretreated with each of the three peptides (Figure 1(c)).

### 3.2. Sea Cucumber-Derived Peptides Reduce the Oxidative Stress Induced by Depletion of Cellular Glutathione

To investigate whether the sea cucumber-derived peptides could modulate natural antioxidant levels in cells, we measured GSH in SH-SY5Y cells. GSH is present in animals and plants and protects cells against a variety of stresses, including oxidative stress, and the GSH level is a good indicator of the health of a cell. We tested the effect of oxidative stress on the amount of GSH in live SH-SY5Y cells using the fluorogenic dye monochlorobimane [21].

**Figure 2:**
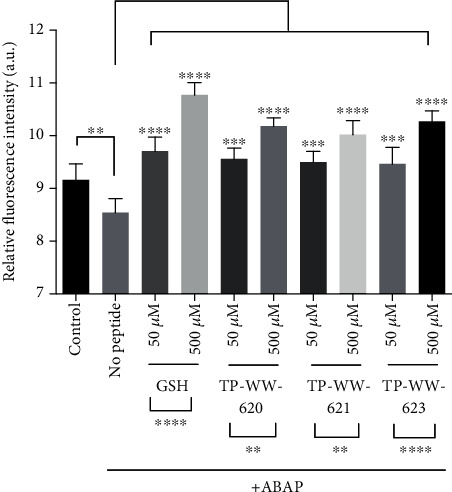
Rescue of depleted GSH levels in stressed cells by sea cucumber-derived peptides and GSH. SH-SY5Y cells were grown in 96-well cell culture dishes until they reached ~80-90% confluency. Cells were then treated with peptides or GSH at 500 *μ*M or 50 *μ*M for 24 h at 37°C, then washed once with PBS before incubation with 40 *μ*M monochlorobimane dye for 1 h in the absence of peptides and GSH at 37°C. Monochlorobimane fluorescence intensity in SH-SY5Y cells was measured using the Envision Multilabel plate reader at Ex/Em 405/486 nm wavelength 1 h after ROS induction with ABAP. “No peptide” represents cells treated with PBS and ABAP whereas “control” means no oxidative stress was induced. For each treatment, four replicates were performed (*n* = 4). Two-way ANOVA and Dunnett's multiple comparison test were applied to measure the statistical significance of each treatment relative to the no-peptide control as well as to compare differences between pairs of peptide concentrations. Statistical significance was determined by Tukey one-way ANOVA. ^∗∗∗∗^*P* < 0.0001, ^∗∗∗^*P* < 0.001, and ^∗∗^*P* < 0.01. Error bars show SD (*n* = 4). Experiments were repeated three times.

Cells grown in 96-well plates were treated with either sea cucumber peptides or GSH at 50 *μ*M and 500 *μ*M concentrations for 24 h at 37°C before washing the cells and incubating them further with 40 *μ*M monochlorobimane at 37°C for 25 min. Afterwards, the cells were treated with 600 *μ*M ABAP (2,2′-azobis(2-amidinopropane)dihydrochloride) to increase the production of ROS [27]. One hour post ABAP treatment, fluorescence intensity in the cells was recorded at Ex^405^/Em^486^ using the Envision Multilabel plate reader (Figure 2). We observed that the application of ABAP significantly reduced the level of GSH in cells treated with vehicle (PBS) whereas cells treated with GSH at both 500 *μ*M and 50 *μ*M concentrations showed significantly increased levels of intracellular GSH (Figure 2). Interestingly, all three peptides, at both 50 *μ*M and 500 *μ*M concentrations, were as effective as GSH at increasing the level of GSH in SH-SY5Y cells (Figure 2). Moreover, the effect of the peptides was dose-dependent. In addition to this, we also evaluated whether the peptides can reduce ROS formation in H_2_O_2_-treated SH-SY5H cells. Results in Figure S1. demonstrate that all three peptides display antioxidant activities in cells, which significantly reduce ROS formation measured by the general oxidative stress indicator CM-H2DCFDA. Together, these data suggest that the sea cucumber-derived peptides can reduce oxidative stress in human neuroblastoma cells.

### 3.3. Sea Cucumber-Derived Peptides Alleviate Oxidative Stress-Induced Mitophagy by Reducing the Level of Superoxide in Mitochondria

**Figure 3:**
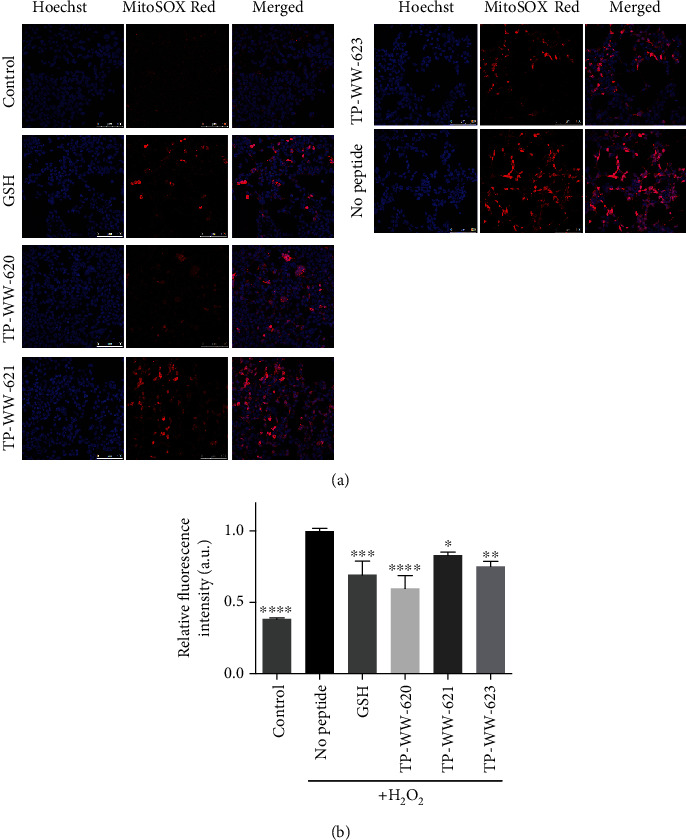
Sea cucumber-derived peptides reduce superoxide levels in mitochondria. (a) Detection of mitochondrial ROS by MitoSOX using confocal microscopy: SH-SY5Y cells were grown in glass-bottomed MatTek dishes until they reached partial confluency (50-70%). Cells were then treated with 50 *μ*M peptides and were incubated for 24 h at 37°C. As a positive control, cells were also treated with 50 *μ*M glutathione (GSH). After incubation, cellular oxidative stress was induced by treating cells with 2 mM H_2_O_2_ for 3 h at 37°C. Cells were then washed three times with PBS before labelling them with 2 *μ*M MitoSOX Red dye for 30 min at 37°C. MitoSOX Red fluorescence intensity was then detected by confocal microscopy. (b) Detection of mitochondrial ROS by flow cytometry: cells were grown and treated as described in (a) except that the cells were cultured in 24-well plates until they reach ~80% confluency. After incubation with MitoSOX Red, cells were washed with PBS and harvested for fluorescence measurements by flow cytometry. In the bar diagram, “control” refers to the negative control where ROS are not induced, and “no peptide” represents the positive control where cells were treated with H_2_O_2_ in culture medium without any peptide treatment. Samples were compared with “no peptide,” and statistical significance was determined by Tukey one-way ANOVA. ^∗∗∗∗^*P* < 0.0001, ^∗∗∗^*P* < 0.001, ^∗∗^*P* < 0.01, and ^∗^*P* < 0.05. Error bars show SD (*n* = 4). Experiments were repeated 3 times.

Oxidative stress can cause functional abnormalities in cell organelles. Therefore, we tested the antioxidant effect of the sea cucumber-derived peptides on mitochondria, as mitochondria are a major source of ROS production in cells [28]. The superoxide level in mitochondria, which is a good indicator of oxidative stress, was measured using the cell-permeable fluorogenic dye, MitoSOX Red [29], in live SH-SY5Y cells. MitoSOX Red targets mitochondria and is readily oxidised to generate red fluorescence. In this assay, SH-SY5Y cells grown in glass-bottomed MatTek dishes were treated with either 50 *μ*M peptides or 50 *μ*M GSH and were incubated for 24 h at 37°C. Post incubation, oxidative stress was imposed by treating cells with 2 mM H_2_O_2_ for 3 h at 37°C, after which cells were washed three times with PBS before labelling with 2 *μ*M MitoSOX Red dye for 30 min at 37°C. Subsequently, cells were imaged using a confocal microscope to observe the changes in the fluorescence intensity of MitoSOX Red (Figure 3(a)). We show that treating cells with H_2_O_2_ alone enhanced the fluorescence intensity of MitoSOX Red, whereas this fluorescence was reduced in cells treated with GSH. The increase of the superoxide level by the addition of H_2_O_2_ is related to the fact that H_2_O_2_ rapidly reacts with oxidant scavengers and therefore leads to higher yield of “leaky” electrons from incompletely reduced oxygen that can subsequently interact with molecular oxygen to form the superoxide anion. Interestingly, we observed an apparent reduction in the fluorescence of MitoSOX Red in cells treated with each of the three peptides (Figure 3(a)). To complement the imaging assay, we performed flow cytometry to quantify MitoSOX Red fluorescence intensity in cells. Cells were treated as described for the imaging assay above, including labelling with MitoSOX Red dye. Cells were then washed with PBS, harvested by trypsinisation, and resuspended in PBS for subsequent flow cytometry analysis. Consistent with the imaging results, quantification by flow cytometry revealed that GSH as well as all three sea cucumber peptides significantly blocked the H_2_O_2_-induced increase in the fluorescence intensity of MitoSOX Red (Figure 3(b)). This suggests that the peptides were able to counteract the increase in superoxide levels within mitochondria.

**Figure 4:**
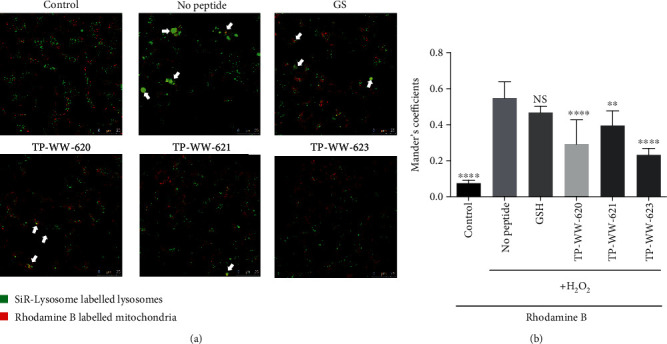
Sea cucumber-derived peptides reduce mitophagy induced by oxidative stress. (a) SH-SY5Y cells were grown in glass-bottom MatTek dishes until they reached partial confluency (50-70%). Next, cells were treated with 50 *μ*M peptides or 50 *μ*M GSH. Peptides were added together with SiR-Lysosome, a lysosomal marker. 24 hours after incubation, cellular oxidative stress was induced by treating cells with 1 mM H_2_O_2_ for 2 h at 37°C. Cells were then washed three times with PBS before the addition of rhodamine B (a mitochondrial stain) for 30 min at 37°C. After a further wash, SiR-Lysosome and rhodamine B fluorescence were detected by confocal microscopy. White arrows point to the mitochondria colocalised with lysosomes. (b) Quantification of the colocalisation of mitochondria and lysosomes was done by measuring Manders' coefficient. Samples were compared with “no peptide,” and statistical significance was determined by Tukey one-way ANOVA. ^∗∗∗∗^*P* < 0.0001, ^∗∗∗^*P* < 0.001, ^∗∗^*P* < 0.01, and ^∗^*P* < 0.05; NS: not significant. Error bars show SD (*n* = 4). Experiments were repeated three times.

It is well established that oxidative stress causes mitochondrial dysfunction and triggers mitophagy [30]; therefore, mitophagy is a subsequent event of oxidative stress and leads to a more severely damaged state of the cell if the excess oxidants cannot be reduced. Defective or damaged mitochondria are removed by cells in a process known as mitophagy, in which the autophagy system ultimately sequesters the mitochondria into lysosomes where they will be degraded. Since the peptides can reduce superoxide levels in mitochondria, we investigated whether they could alleviate oxidative stress-induced mitophagy. To examine the degree of mitophagy in cells, we labelled mitochondria with rhodamine B and lysosomes with SiR-Lysosome (a cathepsin D-specific peptide labelled with silicon rhodamine).  Figure 4(a) shows the distribution of lysosomes and mitochondria in the cytoplasm. In the absence of induced oxidative stress, only 10% of mitochondria (red) colocalise with lysosomes (green) (Figure 4(a)), as quantified using Manders' coefficient analysis (Figure 4(b)). However, upon treatment of cells with H_2_O_2_, ~60% of mitochondria colocalised with lysosomes (Figure 4), suggesting a marked increase in mitophagy. The percentage of mitochondria colocalising with lysosomes was reduced, although not significantly, to ~50% when cells were pretreated, 24 h before exposure to H_2_O_2_, with the natural antioxidant GSH (Figure 4(a)). However, all three peptides significantly reduced the level of mitophagy (Figure 4). The protective effects of peptides on cells were further investigated by evaluating the level of apoptosis and necrosis in H_2_O_2_-treated cells (Figure S3). Oxidative stress induced by H_2_O_2_ can induce 35% of cells to undergo apoptosis and 17% necrosis. We show in Figure S3 that programmed cell death can largely be reduced by the pretreatment of cells with sea cucumber peptides.

### 3.4. Sea Cucumber-Derived Peptides Enter Cells via Both Endocytosis and Diffusion

**Figure 5:**
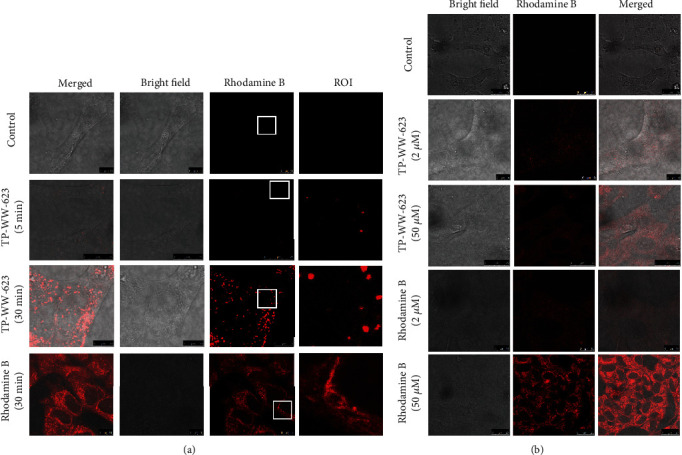
Cellular uptake of rhodamine B-labelled peptides. (a) Confocal imaging of SH-SY5Y cells incubated with either 2 *μ*M or 50 *μ*M rhodamine B-labelled peptide TP-WW-623 for 5 to 30 min at 37°C to show the cellular uptake mechanism. The ROI (region of interest) panel represents zoomed images from the marked squares in the panels labelled “rhodamine B.” (b) SH-SY5Y cells were incubated at 4°C for 15 min to block endocytosis before incubating them with either rhodamine B-labelled TP-WW-623 or free rhodamine B for a further 10 min at room temperature. These cells were then washed three times with PBS before imaging. Experiments were repeated twice.

Although the antioxidant activity assays performed in this study suggest that the peptides are taken up by live cells, we attempted to demonstrate their cellular uptake directly using imaging methods. To achieve this, we stably labelled the peptides with the fluorescent dye rhodamine B. SH-SY5Y cells were then treated with the labelled peptides and were incubated at 37°C for varying lengths of time. As a control, cells were also treated with free rhodamine B dye alone. Confocal microscopy revealed that within 5 min, a proportion of the labelled peptides in the medium had already been internalised in intracellular vesicles (Figure 5(a)). Within 30 min, there was a large increase in the number of intracellular vesicles displaying an increased fluorescence intensity, which suggests that endocytosis represents the major internalisation route for the labelled peptides. However, we also observed weak cytosolic fluorescence in these cells (Figure 5(a), ROI), which indicated that a small fraction of labelled peptides could diffuse into the cytosol by either passing through the cell membrane or by being released from endocytic vesicles after uptake from the extracellular space. To further assess peptide diffusion through the cell membrane in the absence of endocytosis, we incubated SH-SY5Y cells at 4°C, which depolymerises microtubules and thereby blocks endocytosis [31, 32], prior to adding labelled peptides and then imaging the cells by confocal microscopy. Under these conditions, a prominent and homogeneous cytosolic fluorescent signal was observed. The intensity of this fluorescence signal increased with the concentration of labelled peptides (Figure 5(b)) which suggests that the peptides can diffuse into cells through the plasma membrane. In the control experiment, free rhodamine B was diffused rapidly into the cells at both 37°C and 4°C and localised to mitochondria, consistent with previous reports [33, 34].

**Figure 6:**
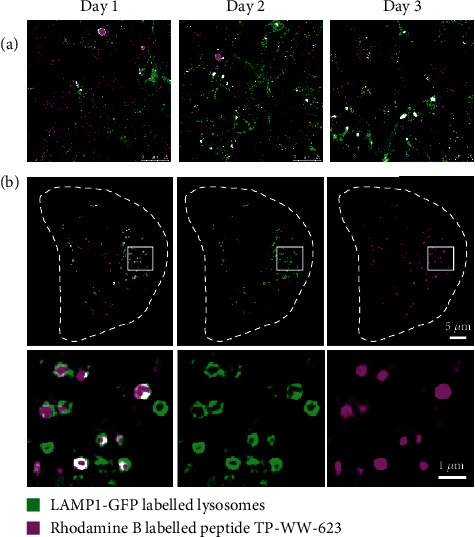
Rhodamine B-labelled peptide TP-WW-623 colocalises with lysosomes. (a) Lysosomes in SH-SY5Y cells were labelled with LAMP1-GFP before incubation with 50 *μ*M rhodamine B-labelled peptide TP-WW-623 at 37°C. Cell images were recorded on three consecutive days to observe the colocalisation of peptides and lysosomes (see also Figure S2). (b) Images of cells recorded by SIM at day 3 to illustrate the colocalisation of peptides and lysosomes. White dashed lines indicate the cell boundary. Enlarged views of the white boxed regions are shown in the bottom panel (see also Figure S2 and Video 1).

Molecules taken up by cells via endocytosis are either recycled back to the cell surface or further processed in late endosomes or lysosomes [35]. To verify whether the labelled peptides are transported to lysosomes via the endocytic pathway, we labelled lysosomes with LAMP1-GFP and then treated these cells with rhodamine B-labelled peptides. Time-course imaging showed that the majority of each labelled peptide rapidly localised within lysosomes and saturated by day 3 after peptide addition (Figure 6 and Figure S2). Further investigation of the internalised peptides by SIM revealed that the peptides are enriched inside the lysosome lumen (Figure 6(b) and Video 1).

### 3.5. Sea Cucumber-Derived Peptides Improve Survival of *C. elegans* Exposed to Paraquat

**Figure 7:**
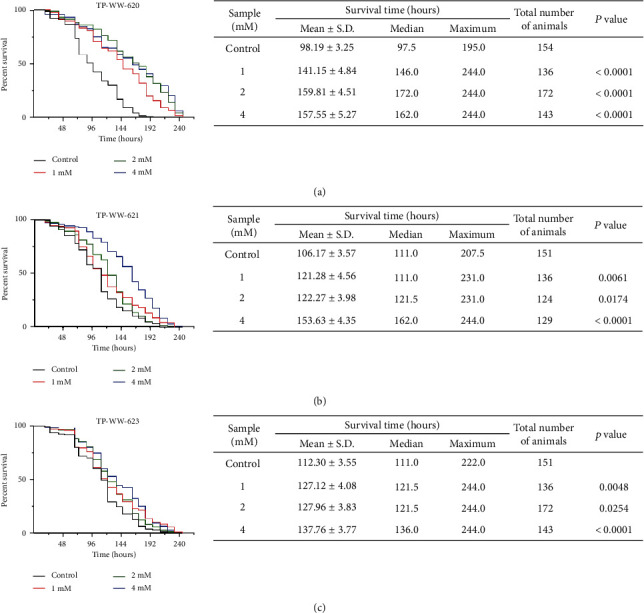
Sea cucumber-derived peptides improve the survival of *C. elegans* exposed to paraquat. Synchronized wild-type L1 nematodes were fed with *E. coli* NA22 and incubated for 42 h. The L4 nematodes were then washed and transferred to 96-well plates (15-20 nematodes per well; >100 nematodes for each treatment) containing *E. coli* NA22 (OD_570 nm_ = 0.5) and peptide samples. After 24 h, the nematodes were exposed to 100 mM paraquat and the numbers of live nematodes were scored under a light microscope every 12 h. Representative Kaplan-Meier survival curves are shown for the nematodes treated with or without indicated concentrations of peptide samples TP-WW-620 (a), TP-WW-621 (b), and TP-WW-623 (c). Tables on the right panel show the quantification of the survival time of paraquat-intoxicated *C. elegans* at different conditions.

Our experiments in live cells demonstrate that the three peptides tested have antioxidant activity and that they can reduce the toxicity of oxidative stress, even at the organellar level. We next wanted to investigate whether these peptides were equally active in alleviating toxicity from oxidative stress in whole organisms. To achieve this, we used the nematode, *C. elegans*, a model organism widely used to study ageing and age-related disorders [36, 37]. Survival in *C. elegans* is severely compromised by exposure to paraquat, which is a toxic oxidant that produces excessive superoxide anions in cells [24, 38]. We tested whether the peptides alleviated paraquat toxicity in *C. elegans* by measuring nematode survival rates. Synchronized L1-stage wild-type worms were grown in 96-well dishes (approximately 25-30 worms per well) at 20°C until the L4 stage. The L4 worms were incubated with the peptides at final concentrations of 1.0, 2.0, and 4.0 mM in S medium in 96-well plates (approximately 15-20 worms per well) at 20°C for 24 h prior to treatment with 100 mM paraquat at 20°C for 24 h. Then, the live and dead worms were scored every 12 h until all the worms were dead. Analysis of survival rates showed that, compared to paraquat-treated controls, treatment with the peptides increased worm survival to varying degrees, with TP-WW-620 performing best (Figure 7). These observations in *C. elegans* complement our results from cellular assays in neuroblastoma cells and further validate the antioxidant activity of the peptides.

## 4. Discussion

A major challenge in adapting natural resources for medicinal or general health products is their complexity as a mixture of diverse components, which complicates the detailed characterisation of their bioactivity [39]. In the current study, we addressed the first of these issues by synthesising individual peptide sequences initially identified in complex protein hydrolysates of the sea cucumber *A. japonicus*. This allowed us to address the second issue without concerns over which components of a complex mixture were responsible for any associated biological activity. We were thus able to test the antioxidant activity of three individual peptides using various experimental approaches in a human cell line and in the nematode *C. elegans*. Our results reveal that all three peptides, selected from a previous screen for potent antioxidant candidates [19], reduce cellular toxicity induced by H_2_O_2_ treatment (Figure 1(c)) and support the function of GSH under oxidative stress conditions (Figure 2). We also demonstrated that the sea cucumber-derived peptides were active at the organelle level, as they reduce superoxide levels in mitochondria, thereby preventing mitophagy, as evidenced by the reduced lysosomal engulfment of mitochondria in peptide-treated cells. Furthermore, cellular uptake experiments (Figure 5) revealed that a major fraction of the peptides was taken up by cells via endocytosis and was entrapped in lysosomes (Figure 6). Although lysosomes are well known as the final destination of endocytosed materials for degradation, it has been reported that small peptides can be secreted from lysosomes [40, 41]. In addition to this, we also detected a small fraction of the peptides in the cytoplasm (Figure 5), which was presumably taken up by passive transport through the plasma membrane and/or released from endocytic vesicles. In a final experiment, we show that the peptides can not only alleviate oxidative stress in cell models but also improve the survival rate of *C. elegans* exposed to paraquat.

The antioxidant activity of GSH is attributed to its cysteine residue, whose thiol group is susceptible to oxidation and thus acts as a reducing agent [42, 43]. The amino acids which have been evaluated as antioxidants, other than cysteine, are methionine, tyrosine, histidine, tryptophan, and phenylalanine [44–46]. Our synthesised peptides contain one or more of these amino acids (Figure 1(b)) and thus play an essential role in the antioxidant activity measured by our assays. Therefore, similarly to GSH, the synthetic peptides may have a direct biochemical effect, acting as reducing agents to protect cells from oxidative stress.

In addition to this direct reduction of ROS, another possible mechanism for peptide function could be via eliciting a cell signalling response that regulates cellular oxidative stress. In response to oxidative stress, the main signalling pathways activated in cells are the mitogen-activated protein kinase (MAPK) signalling cascades, the phosphoinositide 3-kinase (PI3K) or Akt pathway, nuclear factor NF-*κ*B signalling, p53 activation, and the heat shock response [47]. A plausible scenario is that, when cells are treated with synthetic peptides prior to the induction of oxidative stress, the above signalling pathways are activated, which leads to an increase in cell resilience such that they are better able to withstand oxidative stress when it occurs. This is consistent with established antioxidant drug screening strategies where, for example, candidate selection is based upon the ability to induce HSF1 expression in cells [48]. Our data showing that the peptides can reduce H_2_O_2_-induced mitophagy (Figure 4) suggests activation of the Akt signalling pathway, which is a crucial and active pathway in mitophagy [49, 50]. The latter result is also consistent with our nematode experiments where we show that the effects of paraquat exposure, which causes mitochondrial dysfunction and decreases the survival of *C. elegans*, can be reduced by pretreating worms with peptides (Figure 7). Involvement of signalling pathways could be tested by using pharmacological inhibitors or activators of, for example, the Akt pathway, along with peptide treatment of cells under oxidative stress conditions [51, 52]. Therefore, it would be interesting to investigate whether the peptides can activate cellular response pathways to defence oxidative stress in the future.

Collectively, we have shown that certain sea cucumber-derived peptides possess antioxidant activity at both cellular and organismal levels and also provided some insight into their underpinning mode of action, including mechanisms of uptake, reduction of mitochondrial superoxide levels, and alleviation of mitophagy. Sea cucumbers are used traditionally both as health food and therapeutics in China, Japan, and other countries, where they are believed to delay ageing, enhance memory, and counteract fatigue.

Our findings demonstrate that the peptides have potential for use as nutritional supplements for age-related conditions as well as general health maintenance and also validate a model strategy for the discovery of novel bioactive compounds in natural source material.

## Data Availability

All data needed to evaluate the conclusions in the paper are present in the paper and/or the Supplementary Materials. Additional data related to this paper may be requested from the corresponding author.

## Supplementary Materials

Supplementary Materials Figure S1: sea cucumber-derived peptides reduce ROS in H_2_O_2_-treated cells. SH-SY5Y cells were grown in 96-well cell culture dishes until they reached 60-70% confluency. Cells were treated with 50 *μ*M peptides and were incubated for 24 h at 37°C. As a positive control, cells were also treated with 50 *μ*M glutathione (GSH). After incubation, cellular oxidative stress was induced by treating cells with 2 mM H_2_O_2_ for 3 h at 37°C. Cells were then washed three times with PBS before labelling them with 1 *μ*M CM-H2DCFDA for 30 min at 37°C. Fluorescence intensity in SH-SY5Y cells was measured using the Envision Multilabel plate reader at Ex/Em 492/527 nm wavelength. Statistical significance was determined by Tukey one-way ANOVA. ^∗∗^*P* < 0.01. Error bars show SD. Experiments were repeated three times. Figure S2: sea cucumber-derived peptides colocalise with lysosomes. Lysosomes in SH-SY5Y cells were labelled with LAMP1-GFP before incubation with 50 *μ*M rhodamine B-labelled peptides TP-WW-620, TP-WW-621, and TP-WW-623 at 37°C. Shown are images of cells recorded on day 3 to illustrate the level of colocalisation of peptides and lysosomes. Figure S3: sea cucumber-derived peptides protect against apoptotic and necrotic cell death. SH-SY5Y cells were grown in Lab-Tek dishes until they reached 60-70% confluency. Cells were treated with 50 *μ*M peptides and were incubated for 24 h at 37°C. As a positive control, cells were also treated with 50 *μ*M glutathione (GSH). After incubation, cellular oxidative stress was induced by treating cells with 2 mM H_2_O_2_ for 3 h at 37°C. Cells were then washed three times with PBS before labelling them with 1 *μ*l Apopxin Green Indicator for apoptosis assay, or 1 *μ*l 7-AAD for necrotic assay, for 30 min at 37°C before imaging according to the manufacturer's protocol. 200 cells were counted for each condition in each experiment for three repeats. Statistical significance was determined by Tukey one-way ANOVA. ^∗∗∗∗^*P* < 0.0001, ^∗∗^*P* < 0.001, and ^∗∗^*P* < 0.01. Error bars show SD. Experiments were repeated three times. Video 1: live cell imaging by SIM demonstrates the dynamic motions of peptides inside lysosomes. Peptides (magenta) internalised inside lysosomes (green) in an SH-SY5Y cell expressing LAMP1-GFP were imaged over 45 s at 1.5 s/frame.
