# Supplementary material for: Sea Cucumber-Derived Peptides Alleviate Oxidative Stress in Neuroblastoma Cells and Improve Survival in C. elegans Exposed to Neurotoxic Paraquat
Source: Oxid Med Cell Longev. 2021 Apr 19;2021:8842926. doi: 10.1155/2021/8842926 (PMC8075690; doi:10.1155/2021/8842926)
Supplement: Supplementary Materials — Figure S1: sea cucumber-derived peptides reduce ROS in H2O2-treated cells. SH-SY5Y cells were grown in 96-well cell culture dishes until they reached 60-70% confluency. Cells were treated with 50 μM peptides and were incubated for 24 h at 37°C. As a positive control, cells were also treated with 50 μM glutathione (GSH). After incubation, cellular oxidative stress was induced by treating cells with 2 mM H2O2 for 3 h at 37°C. Cells were then washed three times with PBS before labelling them with 1 μM CM-H2DCFDA for 30 min at 37°C. Fluorescence intensity in SH-SY5Y cells was measured using the Envision Multilabel plate reader at Ex/Em 492/527 nm wavelength. Statistical significance was determined by Tukey one-way ANOVA. ∗∗P < 0.01. Error bars show SD. Experiments were repeated three times. Figure S2: sea cucumber-derived peptides colocalise with lysosomes. Lysosomes in SH-SY5Y cells were labelled with LAMP1-GFP before incubation with 50 μM rhodamine B-labelled peptides TP-WW-620, TP-WW-621, and TP-WW-623 at 37°C. Shown are images of cells recorded on day 3 to illustrate the level of colocalisation of peptides and lysosomes. Figure S3: sea cucumber-derived peptides protect against apoptotic and necrotic cell death. SH-SY5Y cells were grown in Lab-Tek dishes until they reached 60-70% confluency. Cells were treated with 50 μM peptides and were incubated for 24 h at 37°C. As a positive control, cells were also treated with 50 μM glutathione (GSH). After incubation, cellular oxidative stress was induced by treating cells with 2 mM H2O2 for 3 h at 37°C. Cells were then washed three times with PBS before labelling them with 1 μl Apopxin Green Indicator for apoptosis assay, or 1 μl 7-AAD for necrotic assay, for 30 min at 37°C before imaging according to the manufacturer's protocol. 200 cells were counted for each condition in each experiment for three repeats. Statistical significance was determined by Tukey one-way ANOVA. ∗∗∗∗P < 0.0001, ∗∗P < 0.001, and ∗∗P < 0.01. Error bar [file 8842926.f1.zip › 8842926.f1.docx]

**Sea cucumber-derived peptides alleviate oxidative stress in neuroblastoma cells and improve survival in *C. elegans* exposed to neurotoxic paraquat**

Meng Lu^a,†^, Ajay Mishra^a,1,†^ Chiara Boschetti^a,2^, Jing Lin^b,c^, Yushuang Liu^c^, Hongliang Huang^c^, Clemens F Kaminski^a^, Zebo Huang^b^, Alan Tunnacliffe^a^, Gabriele S Kaminski Schierle^a^*

^a^Cambridge Infinitus Research Centre, Department of Chemical Engineering and Biotechnology, University of Cambridge, West Cambridge Site, Philippa Fawcett Drive, Cambridge, CB3 0AS, United Kingdom

^b^Research Institute for Food Nutrition and Human Health, School of Food Science and Engineering, South China University of Technology, Guangzhou 510640, China

^c^School of Biosciences and Biopharmaceutics, Guangdong Pharmaceutical University, Guangzhou 510006, China

^1^Current address: The European Bioinformatics Institute (EMBL-EBI), Wellcome Genome Campus, Hinxton, Cambridgeshire, CB10 1SD, United Kingdom

^2^Current address: School of Biological and Marine Sciences, University of Plymouth Drake Circus Plymouth Devon PL4 8AA United Kingdom

^†^ These authors contributed equally.

*Corresponding author: gsk20@cam.ac.uk

**Figure S1. Sea cucumber-derived peptides reduce ROS in H_2_O_2_ treated cells.**

SH-SY5Y cells were grown in 96-well cell culture dishes until they reached 60-70% confluence. Cells were treated with 50 μM peptides and were incubated for 24 h at 37˚C. As a positive control, cells were also treated with 50 μM glutathione (GSH). After incubation, cellular oxidative stress was induced by treating cells with 2 mM H_2_O_2_ for 3 h at 37˚C. Cells were then washed three times with PBS before labelling them with 1 μM CM-H2DCFDA for 30 min at 37˚C. Fluorescence intensity in SH-SY5Y cells was measured using the Envision Multilabel plate reader at Ex/Em 492/527 nm wavelength. Statistical significance was determined by Tukey One-way ANOVA. ** - *P* < 0.01. Error bars show SD. Experiments were repeated three times.

**Figure S2. Sea cucumber-derived peptides colocalise with lysosomes.**

**
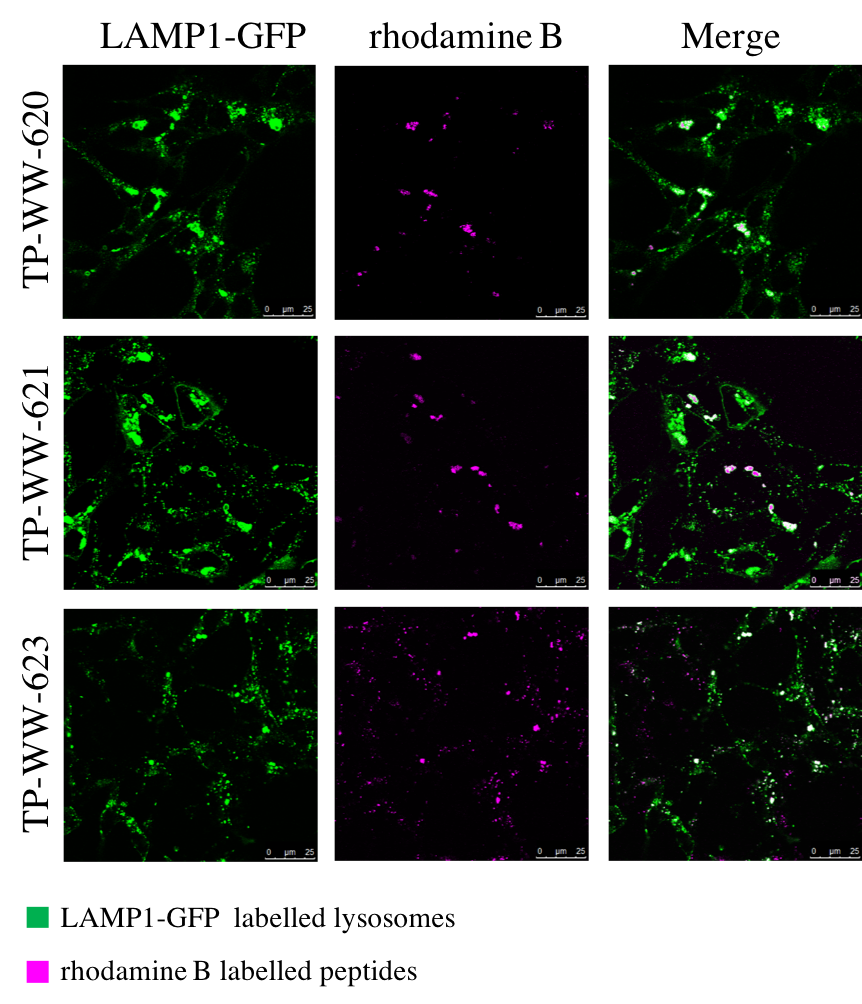
**

Lysosomes in SH-SY5Y cells were labelled with LAMP1-GFP before incubation with 50 µM rhodamine B-labelled peptides TP-WW-620, TP-WW-621 and TP-WW-623 at 37˚C. Shown are images of cells recorded at day 3 to illustrate the level of colocalisation of peptides and lysosomes.

**Figure S3. Sea cucumber-derived peptides protect against apoptotic and necrotic cell death.**

SH-SY5Y cells were grown in Lab-Tek dishes until they reached 60-70% confluency. Cells were treated with 50 μM peptides and were incubated for 24 h at 37˚C. As a positive control, cells were also treated with 50 μM glutathione (GSH). After incubation, cellular oxidative stress was induced by treating cells with 2 mM H_2_O_2_ for 3 h at 37˚C. Cells were then washed three times with PBS before labelling them with 1 μl Apopxin Green Indicator to test for apoptosis, or 1 μl 7-AAD to test for necrosis, for 30 min at 37˚C before imaging according to Manufacturer’s protocol. 200 cells were counted for each condition in each experiment and repeated three times. Statistical significance was determined by Tukey One-way ANOVA. **** - *P* < 0.0001, **- *P* < 0.001, ** - *P* < 0.01. Error bars show SD.

**Video 1. Live cell imaging by SIM demonstrates the dynamic motions of peptides inside lysosomes.**


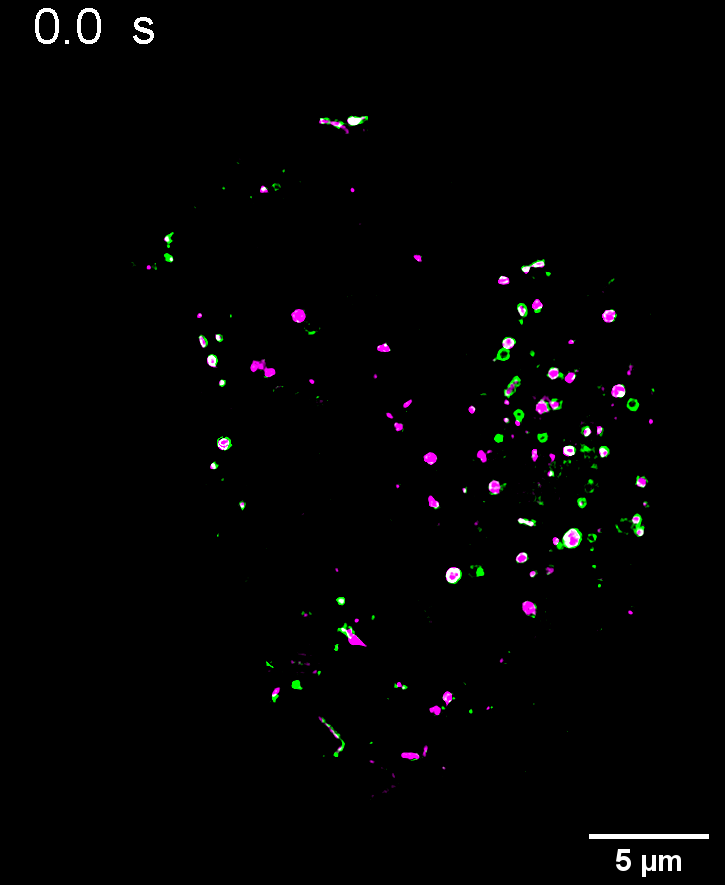


Peptides (magenta) internalised inside lysosomes (green) in an SH-SY5Y cell expressing LAMP1-GFP were imaged over 45 s at 1.5 s/frame.
